# Fabrication and Electric Field-Driven Active Propulsion of Patchy Microellipsoids

**DOI:** 10.1021/acs.jpcb.1c01644

**Published:** 2021-04-20

**Authors:** Jin Gyun Lee, Ahmed Al Harraq, Kyle J. M. Bishop, Bhuvnesh Bharti

**Affiliations:** † Cain Department of Chemical Engineering, Louisiana State University, Baton Rouge, Louisiana 70803, United States; ‡ Department of Chemical Engineering, Columbia University, New York, New York 10027, United States

## Abstract

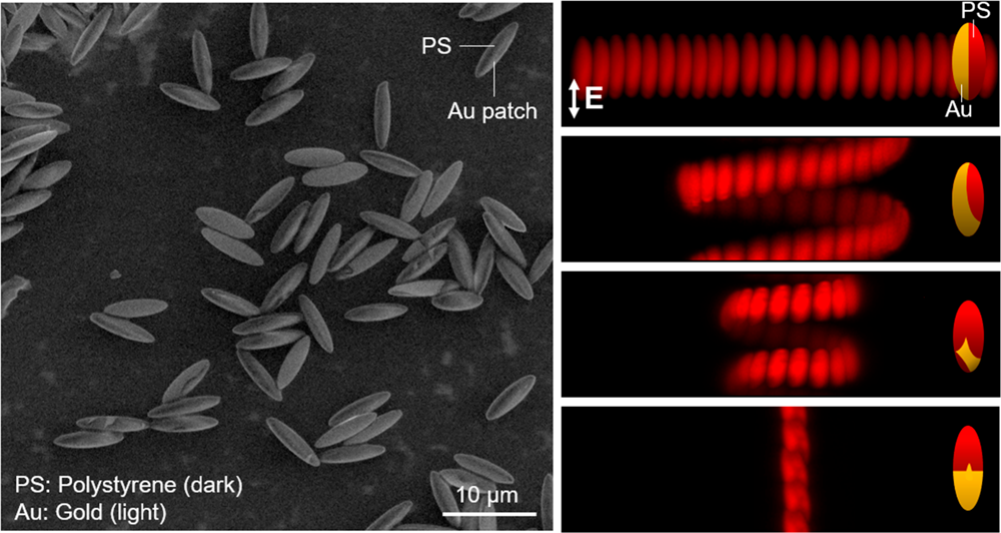

Active colloids are a synthetic analogue of biological microorganisms that consume external energy to swim through viscous fluids. Such motion requires breaking the symmetry of the fluid flow in the vicinity of a particle; however, it is challenging to understand how surface and shape anisotropies of the colloid lead to a particular trajectory. Here, we attempt to deconvolute the effects of particle shape and surface anisotropy on the propulsion of model ellipsoids in alternating current (AC) electric fields. We first introduce a simple process for depositing metal patches of various shapes on the surfaces of ellipsoidal particles. We show that the shape of the metal patch is governed by the assembled structure of the ellipsoids on the substrate used for physical vapor deposition. Under high-frequency AC electric field, ellipsoids dispersed in water show linear, circular, and helical trajectories which depend on the shapes of the surface patches. We demonstrate that features of the helical trajectories such as the pitch and diameter can be tuned by varying the degree of patch asymmetry along the two primary axes of the ellipsoids, namely longitudinal and transverse. Our study reveals the role of patch shape on the trajectory of ellipsoidal particles propelled by induced charge electrophoresis. We develop heuristics based on patch asymmetries that can be used to design patchy particles with specified nonlinear trajectories.

## Introduction

Swimming is a natural method for locomotion across many length-scales, ranging from fish to bacteria.^1^ Movement in fluids is governed by inertial and viscous forces acting on the swimmer; the ratio between these forces is characterized by the Reynolds number.^2^ Locomotion of large organisms such as fish occurs at high Reynolds number where inertial effects override viscous forces.^3^ In contrast, microorganisms swim at low Reynolds number where they cannot rely on inertial forces to move.^4^ Consequently, micron-sized swimmers in biological systems employ several different mechanisms of self-propulsion to navigate through complex environments.^5^ Recent studies have begun to unravel the mechanisms of locomotion at the micron-scale, identifying correlations between the organism shape and its swimming trajectory.^6−8^ For instance, *C. crescentus*, a bacterium widely found in fresh water lakes and streams, is known to swim along helical trajectories in three-dimensions (3D) to enhance motility by tilting its body with respect to its rotating flagellar motor.^9,10^ This enhanced motility is critical in the formation, growth, and survival of the bacterial colonies.^11^

Active colloids are synthetic analogues of microorganisms that mimic the motions of these living objects.^12,13^ In particular, those based on self-phoretic propulsion induce local gradients at the particle surface that drive fluid flows.^14−16^ Particle motion is achieved by introducing physical and/or chemical asymmetries based on particle shape and surface patch characteristics.^17−22^ Engineering particle shape and/or surface patchiness can alter the fluid flows around the particle and is key to directing its motion.^23−26^ The energy source of active colloids is either a chemical reaction or an external field such as magnetic,^27,28^ electric,^17,29,30^ or acoustic.^31,32^ A landmark demonstration of chemically powered swimming is the platinum–gold nanorod in hydrogen peroxide (H_2_O_2_).^33^ This nanomotor was shown to self-propel through solution due to the catalytic decomposition of H_2_O_2_ at the particle surface, resulting in asymmetric ion distributions around the rod. The motion of such chemically powered motors decays over time as the fuel is depleted from the environment.^23^ By contrast, external fields allow for tunable control and enable persistent motion of particles over time by supplying the necessary energy remotely.^23^

It is now well established that the self-propulsion of micro-objects requires the breaking of spherical symmetry to direct translational and rotational motions.^16^ Going beyond this fact to understand the exact relationship between the motion of a particle and its morphological parameters has proven to be difficult. For a given propulsion mechanism, how does the shape and surface anisotropy of the particle determine its trajectory through the fluid? This question is challenging to answer both due to the complexity of the design space of particle shape and patchiness and to our incomplete understanding of the various propulsion mechanisms. Even when these mechanisms are understood, the associated models describing the fluid flows and coupled fields are challenging to solve for the 3D systems of interest, namely, complex particles within structured environments. It is therefore desirable to identify empirical heuristics that can aid in the design of active colloids to achieve desired motions.

Recently, we reported a mechanism for 3D helical motion of spherical particles with preprogrammed surface anisotropy in the form of a triangular metal patch.^34^ Asymmetric fluid flows around such metallodielectric particles in an alternating current (AC) electric field leads to their propulsion in water through a process known as induced charge electrophoresis (ICEP).^29,35−37^ Our previous work helped to demonstrate the link between the motion of an active colloid and the shape of its surface patch. This particular demonstration complements previous investigations on the critical role of particle anisotropy in directing active motions. The challenge at hand is to experimentally observe and correlate asymmetries in both the particle shape and surface patch with characteristics of particle motion such as the diameter and pitch of their helical trajectory.

In this article, we report a simple process for fabricating polymeric ellipsoidal particles decorated with anisotropic metal patches. We use an AC electric field to drive particle motion and correlate features of the anisotropic particles with those of their dynamical trajectories. We choose prolate polystyrene (PS) ellipsoids as model particles due to their well-established fabrication process from PS spheres.^38−40^ We use gold as the metal for the surface patches due to its relatively inert nature and stability in aqueous media. We use physical vapor deposition to form gold patches on ellipsoids previously assembled onto a planar substrate. As the particle packing varies, so does the subsequent patch shape because of the variation in self-shading among the ellipsoids. This fabrication process allows us to create and observe a variety of patch morphologies, which we characterize in terms of patch asymmetries along the transverse and longitudinal axes of the ellipsoids. Upon applying an AC field on the particle dispersion, patchy ellipsoids showcase a variety of motions from linear (perpendicular or parallel to applied field) to nonlinear trajectories as the symmetry of metal patch decreases. We discuss several findings relevant to the programming of ICEP motions of ellipsoids using surface patch morphology. In particular, we describe how the trajectories of patchy ellipsoids can be tailored by varying asymmetries in the patch area along the transverse and longitudinal axes. The heuristics we identify should prove useful in guiding the design of active colloids with programmable motility.

## Materials and Method

### Fabrication of Patchy Ellipsoids

**Figure 1:**
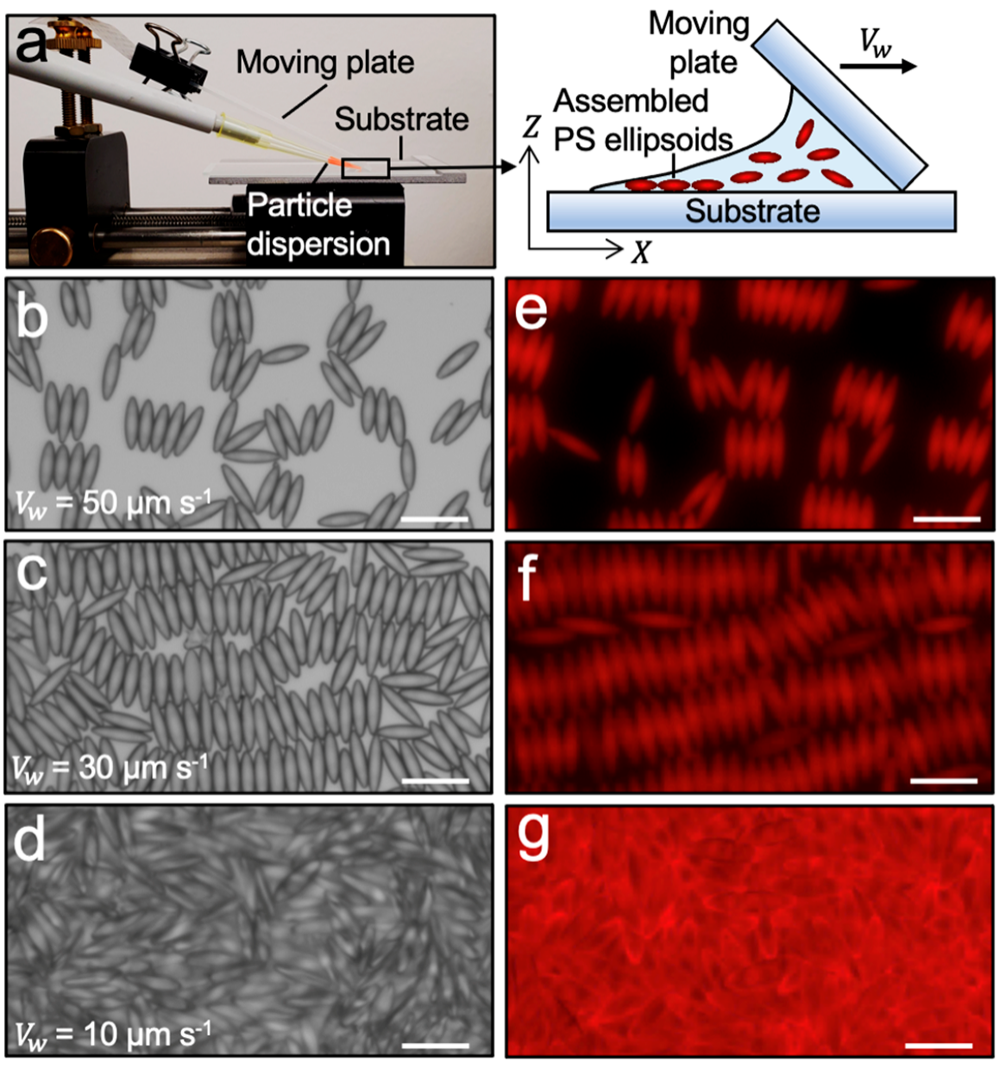
Surface deposition of ellipsoids using the convective assembly method. (a) Photograph (left) and schematic representation (right) of the assembly setup. The constant evaporation of solvent from the dispersion leads to the deposition of particles on the substrate. Brightfield (b–d) and fluorescence (e–g) microscope images of assembled ellipsoids for different speeds of the moving plate (*V*_w_). At a fixed particle concentration and humidity, decreasing *V*_w_ changes the assembly of ellipsoids from submonolayer (*V*_w_ = 50 μm s^–1^) to monolayer (*V*_w_ = 30 μm s^–1^) and multilayer (*V*_w_ = 10 μm s^–1^). Scare bars are 10 μm.

The ellipsoids were prepared by stretching red fluorescent PS microspheres (diameter = 5.1 μm, Magsphere Inc., Figure S1) above the glass transition temperature of the polystyrene.^39^ The spherical particles were prefunctionalized with negatively charged carboxylate groups (zeta potential at pH 7 = −47 mV, Anton Paar Litesizer 500). First, 3.0 g of poly(vinyl alcohol) (PVA) was dissolved in 20 mL of deionized (DI) water by stirring it overnight. Then, 0.40 mL of 2.5 wt % PS beads and 0.60 g of glycerol were added into the PVA solution (PS conc. = 0.05 wt %, glycerol conc. = 3 wt %). The solution was stirred for 2 h and poured into an aluminum tray (dimensions = 10 × 10 cm). After drying the solution for 3 days at 25 °C, a thin PVA film with embedded PS beads was obtained. The film was then placed on a custom-built stretching device and stretched to a desired length (here ∼4 times the original) in the oven at 130 °C. The center-cut of the film was dissolved into a solution of 30 vol % isopropyl alcohol/70 vol % water and washed 10 times by centrifugation before adjusting the final concentration of particles to ∼10 wt % in DI water. The ellipsoids were deposited onto a glass substrate using a convective assembly method (discussed below). The particle dispersion (conc. = 10 wt %, vol. = 10 μL) was added into the gap between a stationary glass substrate (bottom) and a moving glass plate (top). The ellipsoids were assembled by moving the top plate at different speeds at room temperature and relative humidity of 40% (Figure 1a). The assembled ellipsoids were coated with 10 nm of chromium (deposition rate = 0.5 nm s^–1^) followed with 30 nm of gold (deposition rate = 0.1 nm s^–1^) under vacuum (pressure = 1 × 10^–6^ Torr) in a thermal evaporator (Thermionics Laboratory VE-90). For 10 wt % of 10 μL PS ellipsoids dispersion, ∼1.5 × 10^7^ patchy ellipsoids were prepared for each substrate after gold deposition. All experiments were performed with ultrapure water of resistivity = 18.2 MΩ-cm.

### ICEP Experimental Setup

To prepare the coplanar gold electrodes, soda-lime glass slides were soaked in NoChromix solution for 12 h. The glass slides were thoroughly washed with DI water and dried in a convection oven for 6 h. Then, the glass slides with a 2 mm wide paper-mask were coated with 10 nm of chromium followed with 100 nm of gold. The gold-coated ellipsoids were transferred into a vial using a spatula and dispersed in DI water by gentle sonication (particle conc. ∼0.01 wt %). The aqueous dispersion containing patchy particles was transferred to the experimental cell (dimension = 2 mm × 2 mm × 100 μm) made of Teflon tape on the electrode. The coplanar electrodes with a 2 mm gap were connected to a function generator (Agilent) and a high-voltage amplifier (Tegam) using copper tapes. A square wave AC-electric field of magnitude 200 V cm^–1^ at a frequency 10 kHz was applied to the experimental cell. The active motion of the particles was visualized using a light microscope (discussed below). All recorded motions took place at the central region of the experimental chamber between the electrodes to avoid influence of field gradient on the particle dynamics.

### Flow Pattern near Ellipsoids

The fluid flow field around a metal-coated ellipsoid was monitored using nonpatchy polystyrene spheres as tracers. The isotropic tracer particles (diameter = 0.75 μm, Magsphere Inc.) do not self-propel but rather “flow” with the surrounding fluid. The tracers were prefunctionalized with negatively charged carboxylate groups (zeta potential at pH 7 = −38 mV), washed three times with DI water, and dispersed in water at a final concertation of 0.05 wt %. In a typical experiment, gold-coated ellipsoids were immobilized in the experimental cell between the electrodes by drying the particle suspension. After immobilizing the ellipsoids, 10 μL of aqueous dispersion of the tracers was added into the experimental cell. The passive motion of tracers around the immobilized ellipsoids in AC electric field was recorded using a brightfield microscope.

### Microscopy and Particle Tracking

Scanning electron microscopy (SEM) (Quanta 3D DualBeam FEG FIB-SEM) was used to characterize the geometry and assembly of ellipsoids (operating voltage = 1 kV). The substrate with assembled gold-coated ellipsoids was cut into small pieces using a glass cutter for SEM imaging. The surface assembly, patch shape, and active motion of patchy ellipsoids were captured via brightfield and fluorescence microscopy using a Leica DM6 microscope equipped with Texas Red filter cube and EL 6000 fluorescence light source. The active motion of the particles was captured at 50 frames per second using Leica DFC9000 GTC digital camera. The ImageJ software was used to obtain the coordinates of the propelling particles from the experimental videos.^41^ Each video frame of a self-propelling particle was converted to a binary image, from which the particle position was extracted using the “Centroid” feature in “Analyze Particles”. The best fit to each particle trajectory was computed from the kinematic model of eqs 1, 2, and 3 using the Scilab software package (discussed below).

## Results and Discussion

### Surface Assembly and Patch Shape of Ellipsoids

The characteristics of the metal patch fabricated on ellipsoidal particles using metal vapor deposition are governed by their assembly on the substrate. Here, we use a convective assembly method to deposit ellipsoids of average aspect ratio 3.7 on a glass substrate (Figure S2).^42^ In this method, a small aliquot of the particle dispersion is confined in a meniscus between the stationary glass substrate and a moving plate, which is subsequently pushed with a constant speed (*V*_w_). We used a custom-built convective assembly apparatus which employs a conventional syringe pump to push the moving plate with a controllable speed as shown in Figure 1a. For an aqueous dispersion at fixed temperature and relative humidity, the rate of ellipsoid deposition and the corresponding surface pattern formed on the stationary substrate is governed solely by the speed *V*_w_. We study the effect of *V*_w_ on the deposition pattern of PS ellipsoids while their concentrations in the dispersion and the surrounding humidity are fixed. While the moving substrate is pushed at a constant speed *V*_w_, capillary action drags the bulk of the suspension along and leaves behind a thin wet film on the stationary substrate (Figure 1b–g). The flux of liquid compensating for the evaporation of the dispersion leads to the transport of particles to the edge of the wet film. While the film is drying, the particles are attracted to each other by lateral capillary forces, leading to the assembly of the particles on the substrate. The direction of solvent flux pairs with interparticle capillary attraction to impart order on the ellipsoids. Such ordering of particles at fixed temperature and relative humidity is determined by the speed of the moving plate, which provides a useful control parameter. The assembly patterns left after drying are critical in determining the size and shape of the patch formed in the following metal vapor deposition process. In particular, the packing density and the degree of ellipsoid alignment are the two predominant characteristics impacting the morphology of the patch.

We observe three main types of patterns formed by ellipsoids on the glass substrate which are governed by the speed of the moving plate, *V*_w_. For large speeds, that is, *V*_w_ > 50 μm s^–1^, a wet film forms and dries rapidly, leaving behind sparse particle assemblies that form a submonolayer as seen in Figure 1b,e. The fast-moving plate forms a thin film of dilute suspension on the substrate such that particles do not pack closely and assemble mostly due to lateral capillary forces experienced during drying. We observe both tip-to-tip and side-to-side attractions among ellipsoids that lead to their locally ordered arrangements. At intermediate speeds (30 μm s^–1^ < *V*_w_ < 50 μm s^–1^), ellipsoids pack into monolayers or near-monolayers (Figure 1c,f). In this regime, both capillary forces and particle packing effects collaborate to order the particles in a fashion reminiscent of smectic liquid crystals.^43^ This assembly is characterized by a predominance of side-to-side capillary attraction which provides positional order in addition to orientational order.^44^ Below a threshold speed of a moving plate, here *V*_w_ = 10 μm s^–1^, we find that the wet film formed is significantly denser in particles and thicker than their radius. This allows multilayer formation as ellipsoids have just enough space and time to pack tightly. Under these operating conditions, the assembly loses all visible translational order and the effect of convective deposition on orientational order is also weakened.^45,46^ The result is the overlap of layers of ellipsoids, as seen in Figure 1d,g.

**Figure 2:**
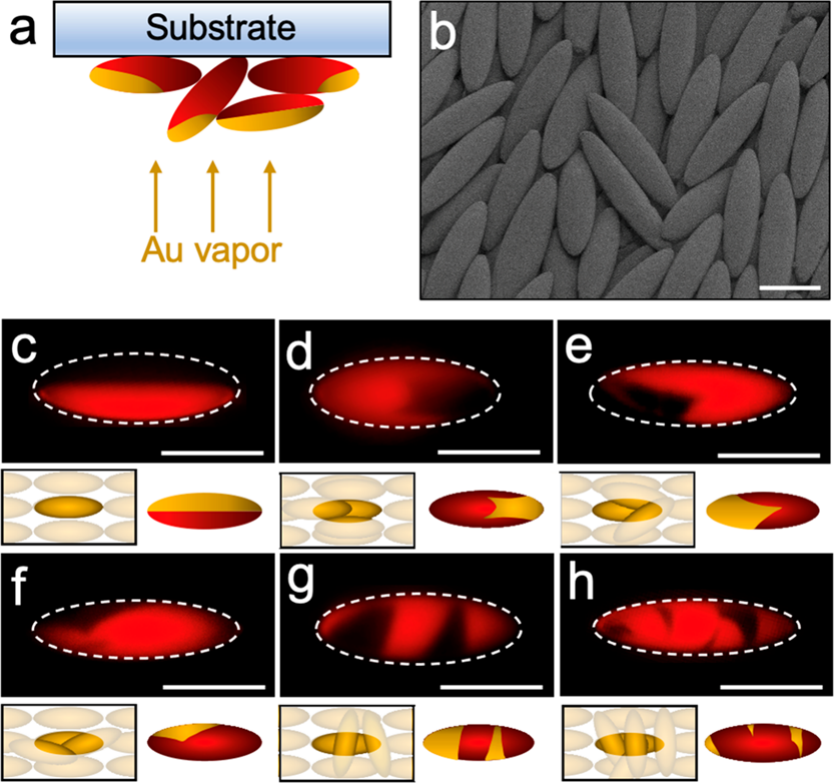
Physical vapor deposition of gold on ellipsoids. (a) Schematic representation of metal vapor deposition on a substrate coated with a layer of ellipsoids. Depending on the characteristics of the layer, the constituent ellipsoids can be self-shaded, leading to the formation of a complex metal patch(es) on the particle. (b) SEM image showing a multilayer of ellipsoids assembled on the substrate. (c–h) Fluorescence microscope images showing the ellipsoids with complex gold patch(es) on their surface. The metal patch(es) appears black on the red PS particle core. The schematic below each fluorescence image represents the arrangement of ellipsoids during the metal deposition process (left) and the resulting patch shape (right). Scale bars are 10 μm.

Glass substrates covered with ellipsoids are transferred to the evaporator for metal vapor deposition (Figure 2a), specifically 10 nm of chromium followed by 30 nm of gold. We find that the shape and symmetry of the metal patch depend on the assembly of ellipsoids on the substrate, which can be directed by the deposition particle speed, *V*_w_. For ellipsoids assembled in a single layer, the resulting patch morphology is the well-known Janus type, in which one hemiellipsoid is coated with metal while the opposite is polymeric.^21,47,48^ In the case of multilayered packing of ellipsoids on the substrate, we find significant overlap among particles as seen in Figure 2b. In this case, metal vapor deposition gives rise to a variety of anisotropic patch shapes and sizes, which are exemplified in Figure 2c–h and Figure S3. Here, the reduced symmetry of patch is a result of neighboring particles that partially cover the surface of an underlying ellipsoid. Adjacent ellipsoids act as shading masks to the underlying particle during vapor deposition, resulting in different shapes of patches within a single batch synthesis.^22,49^ Depending on the number of neighboring particles and their shading effect, the fabricated patchy ellipsoids can have complex surface anisotropy. Multiple overlapping ellipsoids can lead to the formation of a single patch with unusual shape, such as the ones shown in Figure 2d–f. Alternatively, multiple shadings can lead to the formation of multiple patches on the same particle surface, as shown in Figure 2g,h.

### Flow Profile around a Patchy Ellipsoid and Active Propulsion

**Figure 3:**
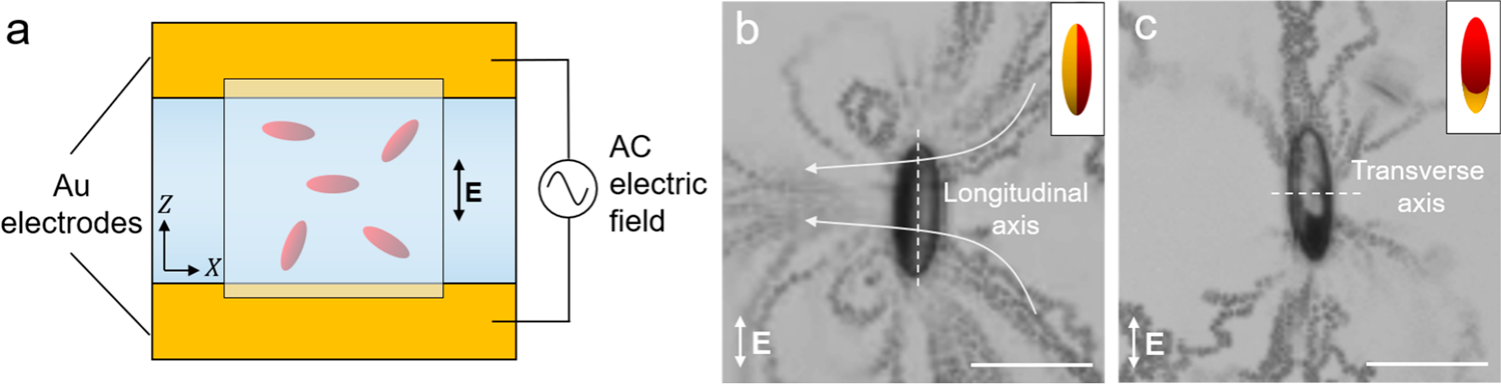
Fluid flow around a metallodielectric patchy ellipsoid. (a) A schematic showing the experimental setup of coplanar electrodes. The trajectory of the active particles is recorded using video microscopy while applying the AC field. (b,c) Superimposed images showing ICEO flow around ellipsoids with a Janus patch (b) and a patch of lower symmetry (c). The dashed lines indicate ellipsoid’s longitudinal (b) and transverse axis (c). The arrows on the images indicate the direction of fluid flow around the particle. The insets in (b,c) are schematics of each patchy ellipsoid. Scale bars in (b,c) are 10 μm.

The application of an electric field to a polarizable surface suspended in a fluid induces charge separation and fluid flow at the surface known as induced-charge electro-osmosis (ICEO).^35,36^ The asymmetric ICEO flows around a patchy ellipsoid provide the driving force for particle propulsion in AC electric fields. Here, we immobilize ellipsoids with different patch shapes at the surface of the experimental cell and visualize the field-induced flows using PS tracer spheres (diameter = 0.75 μm). The fluid flow around each ellipsoid is visualized by recording a video of tracer isotropic PS spheres moving in the flow and superimposing the frames of the video. The field-induced flow around a Janus ellipsoid immobilized on the experimental cell is shown in Figure 3b. The ICEO flow is characterized by an asymmetric pattern about the ellipsoid’s longitudinal axis, showing a pair of vortices near the gold patch indicative of higher velocity on the gold side as compared to the PS side (Figures 3b and S4a). The higher fluid velocity near the metal patch is due to the larger polarizability of the metal surface as compared to PS^20^ (Movie S1). The ICEO flow around a second particle shows additional asymmetries in the flow, reflecting the lower symmetry of the particle (Figures 3c and S4b). As for the Janus particle, the ICEO flow is directed primarily from the PS side of the particle to its metal side. By contrast, however, this primary flow is asymmetric across both the transverse and longitudinal axes for the lower-symmetry particle as shown in Figure 3c and Movie S1. Because of the complex patch shape, there is a net flow oblique to both the longitudinal and the transverse axes of the ellipsoid which induces nonlinear ICEP in solution as we detail below.

To investigate the effect of asymmetric ICEO flow around a patchy ellipsoid on the trajectory of unbound particles, we perform systematic experiments with particles of varying patch shape and size. The experiments were performed in an AC electric field *E* with magnitude of 200 V cm^–1^ and frequency of 10 kHz as detailed in Materials and Methods. Note that the motion of the particles induced by ICEO is termed ICEP (discussed above). The application of the AC electric field orients a patchy ellipsoid with its longitudinal axis parallel to the direction of the field *E*. The reorientation of the particle is due to electric and hydrodynamic contributions to the driving torque.^50,51^ For ellipsoids of aspect ratio 3.7, we find that nearly all particles exhibit such alignment irrespective of patch size and shape (Figure S5). Once aligned in the field, particles move along trajectories which depend on the shape and size of their patch. In the case of Janus ellipsoids, we observe that particles move toward their PS face along linear trajectories directed perpendicular to the applied field (Movie S2). Ellipsoids with anisotropic patches swim along nonlinear trajectories such as circular and helical (Movie S2). No motion is observed for bare ellipsoids without metal patches.

**Figure 4:**
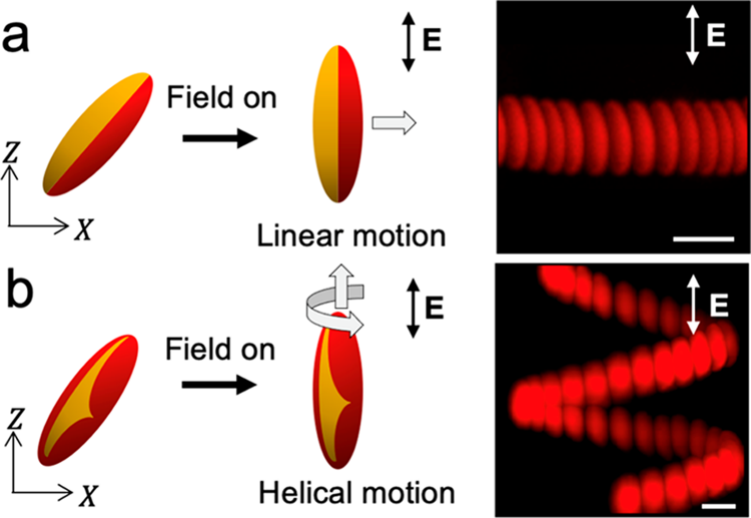
Propulsion of patchy ellipsoids in electric field. Schematic (left) and superimposed images (right) showing the linear propulsion of a Janus ellipsoid (a) and the helical propulsion of a patchy ellipsoid with low symmetry patch (b). Scale bars are 5 μm.

The 1D linear motion of a Janus ellipsoid is attributed to asymmetric fluid-flow between the gold and PS sides of the particle, resulting in the force imbalance normal to the longitudinal axis (Figures 3b and 4a). No motion is observed along the direction of the applied field, consistent with the (approximate) mirror symmetry of the particle and the surrounding flows about a plane normal to the longitudinal axis. Such fluid flow and particle motion is identical to that observed for metallodielectric Janus spheres.^20,29^ For a patchy ellipsoid self-propelling along a helical trajectory, particle translation away from its patch along the transverse axis is augmented by two additional motions, namely, translation along the longitudinal axis and rotation about that axis (Figures 3c and 4b). These motions are attributed to additional contributions to the driving force and torque caused by the patch asymmetry. The asymmetric fluid flow across the ellipsoid’s longitudinal axis drives the rotational motion while the ICEP force imbalance across the transverse axis induces the linear motion along with *Z*-direction.

### Effect of Patch-Symmetry on ICEP Motion of Ellipsoids

**Figure 5:**
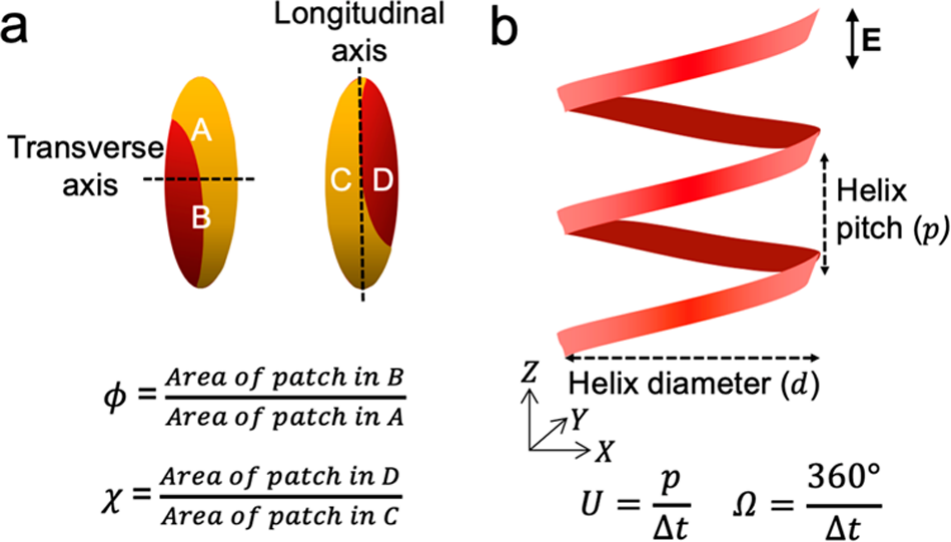
Characterization of active propulsion of patchy ellipsoids in electric field. (b) Definition of the patch ratios characterizing the degree of symmetry along the longitudinal axis (ϕ) and the transverse axis (χ). (a) Schematic representation of helical trajectory. The period (Δ*t*) is the time required for an ellipsoid to travel one complete turn of the helix.

The shape and symmetry of metal patch governs the trajectory of helically swimming particles such as pitch and diameter, and the handedness of helix is dependent on the initial orientation of particles upon application of electric field.^34^ To understand the relationship between the patch shape and the particle trajectory, we analyze the patch of each ellipsoid to quantify asymmetries in the distribution of metal on the PS surface. We first image the particles using fluorescence video microscopy during their helical motion and identify frames in which the particle is closest to the microscope objective. In this way, we capture each particle in a particular orientation about its longitudinal axis as it rotates with each turn of the helix. From these images we determine the patch area by analyzing the “dark” pixels on the particle surface where the gold coating attenuates the fluorescent signal (Figure 2c–h). To quantify patch asymmetries in the longitudinal direction, we first divide the image into two regions separated by the transverse axis (Figure S6 and Figure 5). By convention, the region with the larger patch area is denoted as “A” and that with the smaller patch area as “B”. The longitudinal symmetry parameter ϕ is defined as the ratio between the area of the patch in region B and the area of the patch in region A such that 0 ≤ ϕ ≤ 1. Patch asymmetries in the transverse direction are quantified in similar fashion by dividing the image into two regions denoted as “C” and “D” and separated by the longitudinal axis. The transverse symmetry parameter χ is defined as the ratio between the area of the patch in region D and the area of the patch in region C such that 0 ≤ χ ≤ 1. The values of ϕ and χ are determined by the self-shading of the ellipsoids in the surface layer during the patch fabrication process (discussed above) and by the particle orientation as it moves in the field. We note that the patch area estimated from 2D fluorescence micrographs is a projection of the 3D patch and may vary from the true area of the patch. Nevertheless, the observable ratios ϕ and χ provide convenient and unambiguous features to correlate characteristics of the particles and their trajectories.

**Figure 6:**
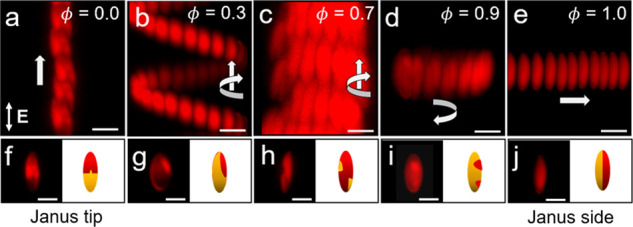
Active propulsion of patchy ellipsoids for different values of the longitudinal symmetry parameter ϕ showing dissimilar particle trajectories (a–e). Superimposed images of patchy ellipsoids self-propelling linearly in the *Z*-direction (a), along helical trajectories (b,c), along circular trajectories (d), and linearly in the *X*-direction (e). (f–j) Fluorescence microscope images (left) and schematic representations (right) of patchy ellipsoids for each corresponding ϕ above. Scale bars are 5 μm.

When the AC field is applied on a Janus ellipsoid with a metal tip (ϕ = 0), the ellipsoid’s longitudinal axis is aligned with the direction of applied field.^50−53^ As a result, the ellipsoid propels linearly along the *Z*-direction (Figure 6a,f). Note that such linear motion along the *Z*-direction is observed in metallodielectric ellipsoids with Janus tip which is not the case with Janus spheres. This motion of ellipsoid is due the “forced” alignment of the particle (and patch) in the direction of applied field due to its elongated shape. Once the ellipsoid attains the aligned configuration, the higher fluid flow on Janus tip leads to the ICEP of particle along the direction of applied field. As the longitudinal symmetry ϕ of the metal patch increases, the patchy ellipsoids swim along helical or circular trajectories (Figure 6b–d and g–i). For maximally symmetric patches with ϕ = 1 such as a Janus ellipsoid, the particle propels linearly along the *X*-direction (Figure 6e,j).

**Figure 7:**
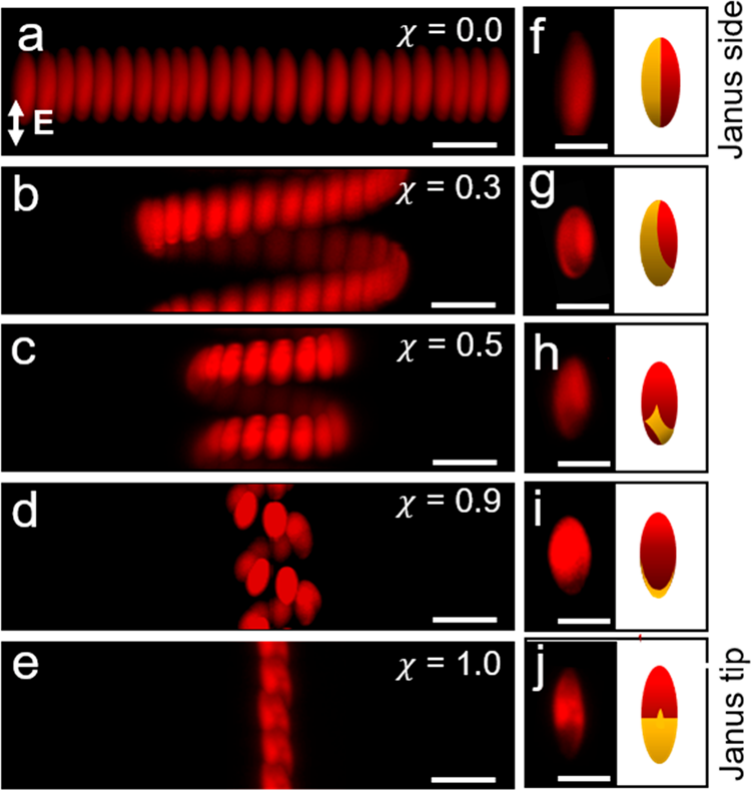
Active propulsion of patchy ellipsoids for different values of the transverse symmetry parameter χ showing different particle trajectories (a–e) Superimposed images of patchy ellipsoids self-propelling linearly in the *X*-direction (a), along helical trajectories (b–d), and linearly in the *Z*-direction (e). (f–j) Fluorescence microscope images (left) and schematic representations (right) of patchy ellipsoids for each corresponding χ above. Scale bars are 5 μm.

Upon increasing the transverse symmetry parameter χ, the patchy ellipsoid changes its trajectory from linear to 3D helical motion (Figure 7). For helically swimming ellipsoids, we find that the helix diameter decreases upon increasing χ (Figure 7b–d and g–i). Finally, a Janus ellipsoid with a metal tip (χ = 1) follows a linear trajectory along the direction of the field (Figure 7e,j). The transition from linear to helical motion (or vice versa) and the changes in features of the helical trajectory such as pitch and diameter appear to correlate with the symmetry parameters χ and ϕ characterizing the patch geometry (discussed below).

For particles moving along helical trajectories, the particle position expressed in Cartesian coordinates (*x*,*y*,*z*) is well approximated by the kinematic equations
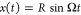
1
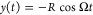
2
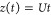
3

Here, *U* is the velocity of the particle along the *Z*-direction parallel to the applied field. The particle rotates steadily about this axis with an angular velocity Ω. At the same time, the particle moves in the *XY*-plane perpendicular to the field with a speed *R*Ω as it traces a circular orbit of radius *R*. Together these motions trace a helix aligned parallel to the applied field with radius *R* and pitch *p* = *U*/Δ*t* where Δ*t* = 2π/Ω is the time required for the particle to complete one full turn. Consistent with our previous work on patchy spheres, we observe that the helix pitch and radius are determined by the particle geometry independent of the applied field strength.^34^ In that study, we used achiral particles with (approximate) mirror symmetry for which the handedness of the helix was determined by the initial orientation of the particle upon application of the field.^34^ By contrast, the majority of patchy ellipsoids reported here lack such mirror symmetry; the handedness of their helical trajectories is likely dependent on the patch shape.

**Figure 8:**
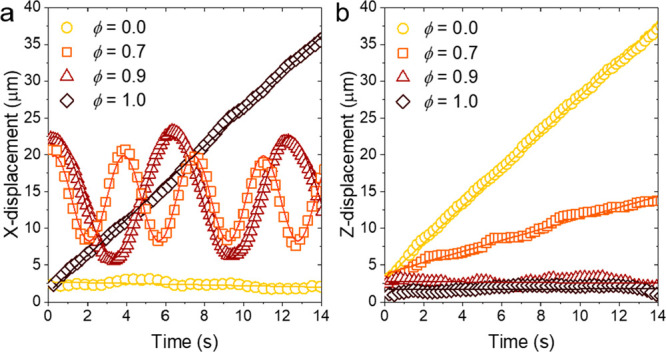
Displacement of patchy ellipsoid in the *X*- and *Z*-directions as a function of time and longitudinal symmetry parameter ϕ. (a) The *X*-location of Janus ellipsoids (ϕ = 0 or 1) over time shows a linear trajectory. For an ellipsoid with an asymmetric patch (0 < ϕ < 1), the oscillating behavior along the *X*-direction in time is a signature of helical motion. (b) As the longitudinal symmetry parameter ϕ increases, the linear velocity of the ellipsoid along the field direction decreases due to the increasingly symmetric fluid flows at the particle. The markers are experimental data for the particle position; the lines represent the best least-squares fit using eqs 1, 2, or 3. All experiments were performed at applied field strength of 200 V cm^–1^ and a frequency of 10 kHz.

For a Janus ellipsoid with a metal tip (ϕ = 0), the speed *R*Ω in the *X*-direction is approximately zero while the particle position in the *Z*-direction increases linearly with speed *U* (Figure 8a,b). Thus, the ellipsoid moves along the direction of the AC field toward one electrode or the other with *U* > 0 or *U* < 0 depending on the initial orientation of the particle. For 0 < ϕ < 0.9, the position of the ellipsoid oscillates along the *X*-direction as it changes steadily in the *Z*-direction as expected for helical motion. For helical motion, we find that the helix pitch is governed by the competition of ICEP forces originating from each hemiellipsoid. As the longitudinal symmetry ϕ increases to approach a Janus particle (ϕ = 0.9), the patchy ellipsoid follows a circular trajectory with oscillatory motion in the *X*-direction with little to no motion in the *Z*-direction. Finally, for a Janus ellipsoid with maximal symmetry along the transverse direction (ϕ = 1), the particle propels along the *X*-direction showing a linear increase in the *X*-displacement with no change in *Z*-displacement. Such linear motion in the *X*-direction with velocity *V* is described by the kinematic model when *U* = 0 and *R*Ω → *V* as *R* → ∞ and Ω → 0.

**Figure 9:**
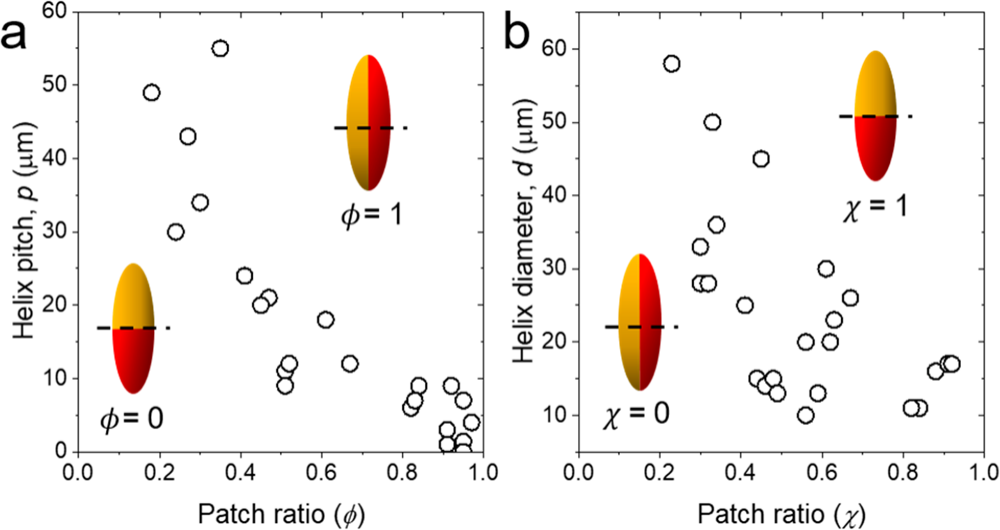
Correlating features of the helical trajectories with those of the metal patches. (a) The pitch of the helix *p* decreases monotonically with increasing longitudinal symmetry ϕ. (b) The helix diameter *d* also decreases with increasing transverse symmetry χ; however, the strength of the correlation is weaker.

In a Janus ellipsoid with a metal side (χ = 0), the particle performs linear motion in the *X*-direction, that is, perpendicular to the applied field. We find that as the transverse symmetry χ increases, the patchy ellipsoid performs a helical motion. Here, the diameter of the helical trajectory decreases upon increasing χ (Figure 9b). For example, upon increasing χ from 0.3 to 0.8, the trajectory of patchy ellipsoid remains as 3D helix while the helix diameter decreases from 50 to 11 μm. As the transverse symmetry of the ellipsoid approaches unity, that is, χ → 1, the particle moves linearly along the *Z*-direction without any motion along the *X*- or *Y*-direction, that is, the particle moves parallel to the applied field *E*.

Our experimental results establish the correlation between patch anisotropy and characteristics of the trajectory of ellipsoidal particles. We acknowledge that the observed experimental correlation between *p* and ϕ is stronger than *d* and χ. The observed lack of strong correlation between *d* and χ may point the potential role of rectified electric field gradients and wall effects on the motion of ellipsoidal particles.^54^ However, current experimental approaches do not allow for resolving the contribution for such effects in the ICEP motion of the particles. Our work does point to the role of surface asymmetry on the propulsion of active particles in external electric fields and lays a foundation to design colloids with tunable trajectories. Note that the helix pitch and diameter may be codependent on ϕ and χ but resolving such codependency based on the experimental data is not possible, primarily because of the lack of precise control over the characteristics of the patch. Further experimental and theoretical work is needed to uncover such relationships.

## Conclusion

**Figure 10:**
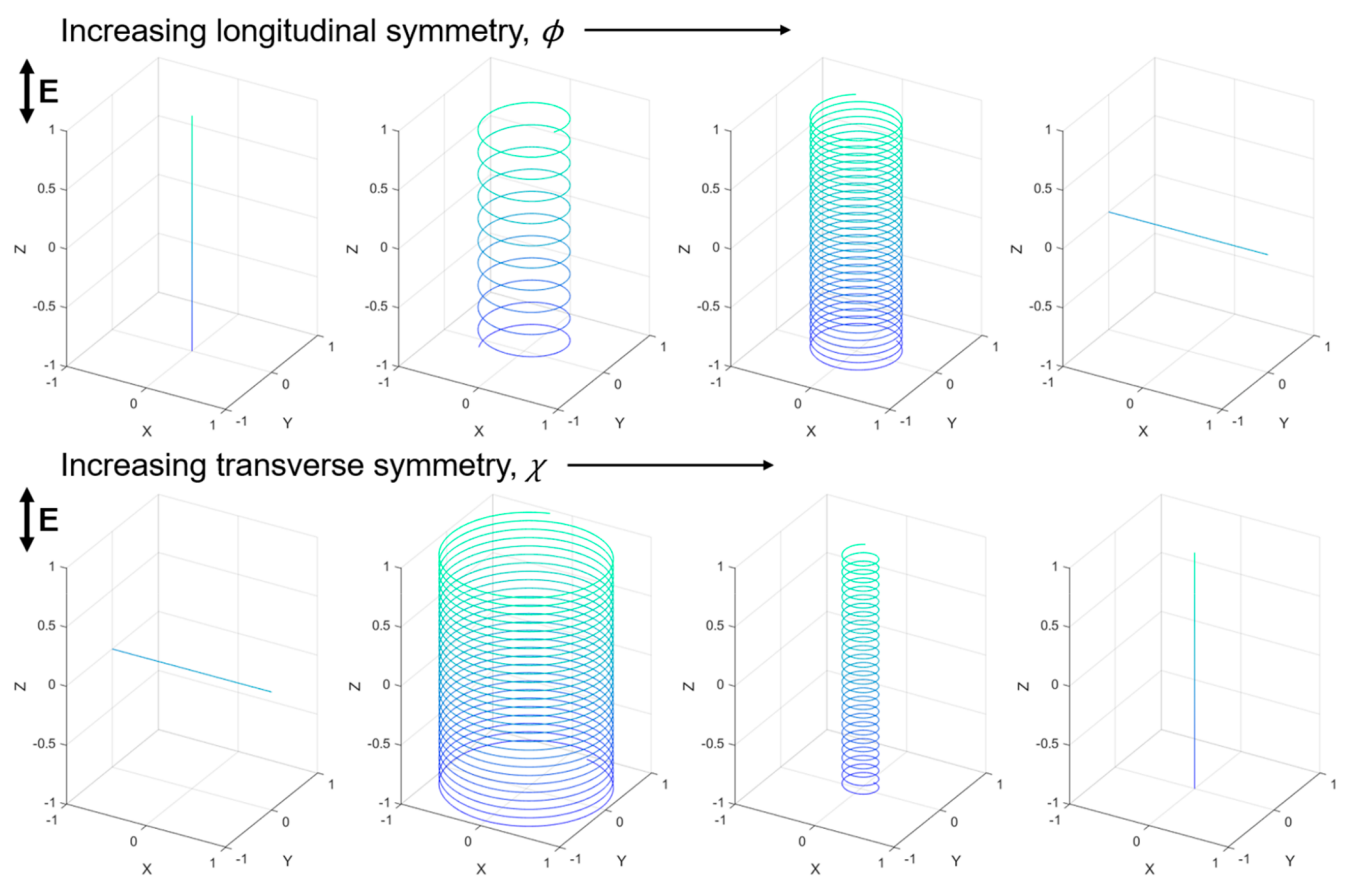
Schematic plots summarizing the effect of longitudinal and transverse patch symmetry of ellipsoidal particles on their active propulsion trajectories.

In summary, we presented a method for the fabrication of PS ellipsoids with anisotropic metal patches and described their active ICEP. The packing of ellipsoids on the substrate can be controlled by deposition speed as solvent evaporation drives their assembly. For multilayered assemblies, ellipsoidal particles can be partially shaded by neighboring particles during the metal vapor deposition, resulting in a wide variety of metal patch shapes on the ellipsoids. When patchy ellipsoids are energized by AC electric field, the particles exhibit complex motions dictated by the shapes of their patches. The dynamic trajectories observed here include the 1D linear motion of Janus ellipsoids in the *X*- and *Z*-directions, 2D circular orbits in the *X*- and *Y*-planes, and 3D helical trajectories. For patchy ellipsoids with helical trajectories, we show that the helix pitch and diameter are correlated to features of the patch describing asymmetries along the particles’ longitudinal and transverse axes (Figure 10). The ability to direct active propulsion using surface patch geometry is not limited to ICEP motions powered by electric fields but can be readily extended to active or driven particles powered by chemical fuels or magnetic fields. This approach provides a general tool for directing the motions of colloidal particles.

## Supporting Information Available

Figure S1, characterization of polystyrene (PS) spheres used for fabrication of ellipsoids; Figure S2, size distribution of PS ellipsoids; Figure S3, SEM images showing the ellipsoids with complex shape of metal patch(es) on the PS surface; Figure S4, superimposed images showing fluid flow profile around metallodielectric ellipsoids with the Janus patch and the patch of lower symmetry; Figure S5, alignment of self-propelling patchy ellipsoids in an electric field; Figure S6, fluorescence microscope images describing the patch ratio along the transverse axis and longitudinal axis of an ellipsoid (PDF)
Fluid profile around a metallodielectric ellipsoid (MP4)
Active motion of a metallodielectric ellipsoid (MP4)
