# Supplementary material for: Fabrication and Electric Field-Driven Active Propulsion of Patchy Microellipsoids
Source: J Phys Chem B. 2021 Apr 20;125(16):4232–40. doi: 10.1021/acs.jpcb.1c01644 (PMC8279480; doi:10.1021/acs.jpcb.1c01644)
Supplement: Supplementary file 1 — jp1c01644_si_001.pdf [file jp1c01644_si_001.pdf]

Supporting Information

Fabrication and Electric Field-driven Active Propulsion of Patchy Microellipsoids

Jin Gyun Lee[a], Ahmed Al Harraq[a], Kyle J. M. Bishop[b], and Bhuvnesh Bharti*[,a]

[a]*Cain Department of Chemical Engineering, Louisiana State University, Baton Rouge, LA 70803*

[b]*Department of Chemical Engineering, Columbia University, New York, NY 10027*

*corresponding authors: bbharti@lsu.edu

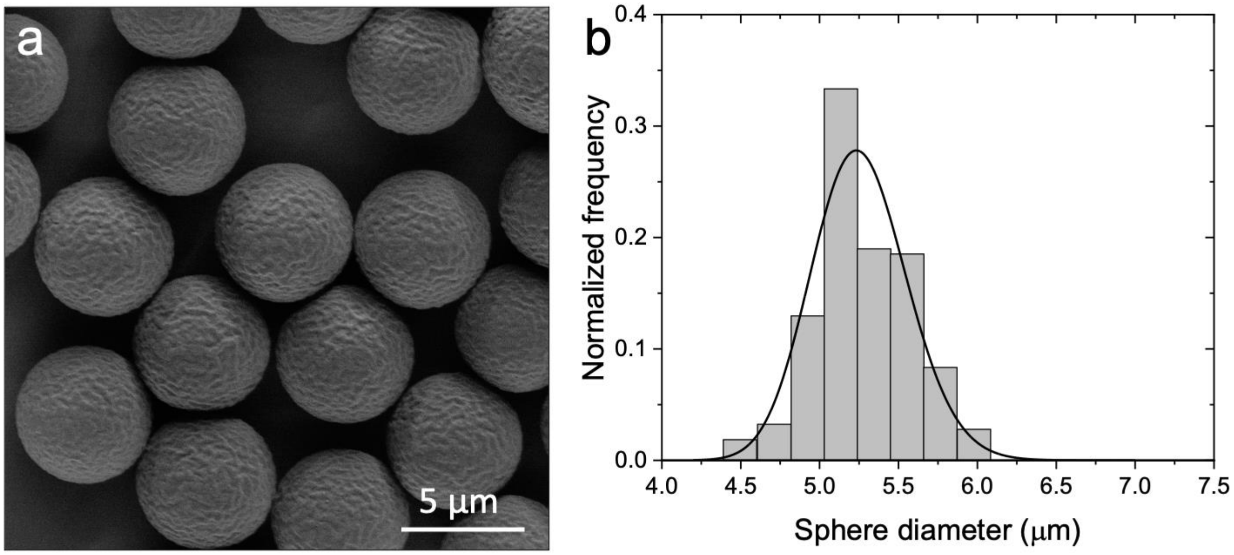

Figure S1. Characterization of polystyrene (PS) spheres used for fabrication of ellipsoids. (a) SEM image of PS spheres. (b) Size distribution of PS spheres showing an average diameter of ~5.1 µm. The size distribution was obtained by image analysis of optical micrographs with a sample size of ~500 spheres using ImageJ software. The bars are the measured diameters, and the line represents the best-fit using log-normal distribution function.

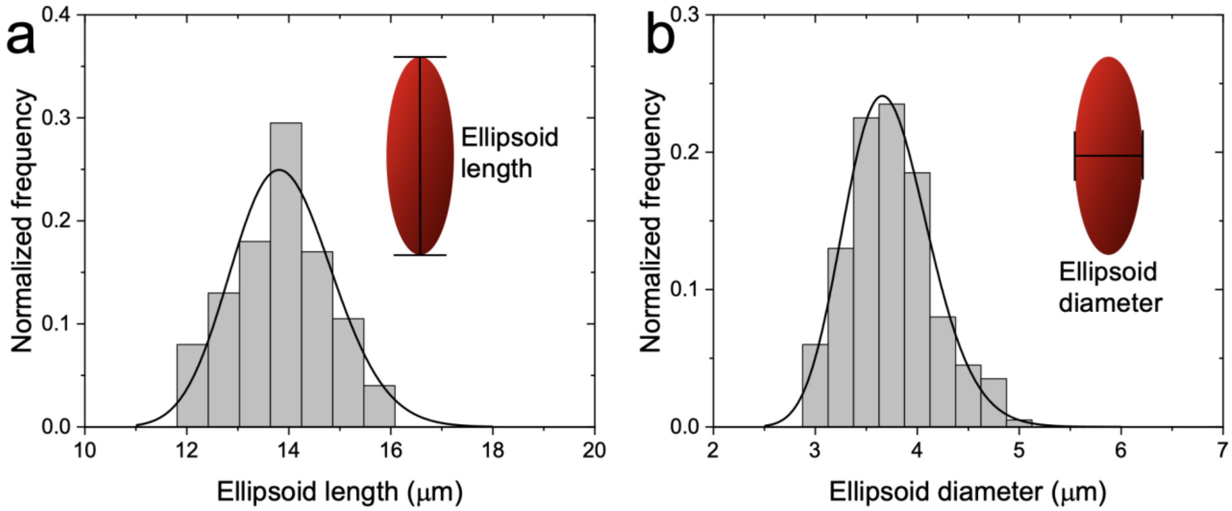

Figure S2. Size distribution of PS ellipsoids. (a) Length distribution of ellipsoids showing an average length of ~13.7 μm. (b) Diameter distribution of ellipsoids showing an average diameter of ~3.7 μm. The size distribution was obtained by image analysis of optical micrographs with a sample size of ~500 ellipsoids using ImageJ software. The bars are the measured data, and the line represents the best-fit using log-normal distribution function.

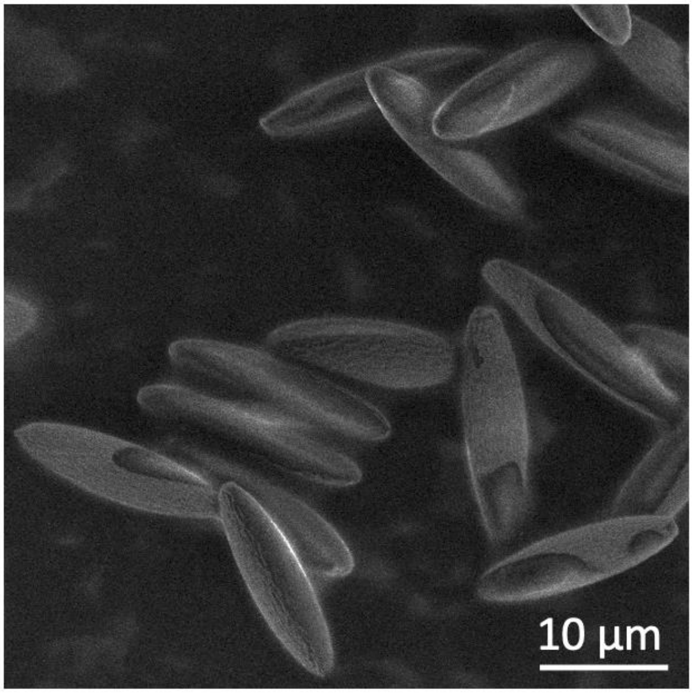

Figure S3. SEM images showing ellipsoids with metal patch(es) on the PS surface. The metal patch(es) appears as light gray, and the PS surface is darker.

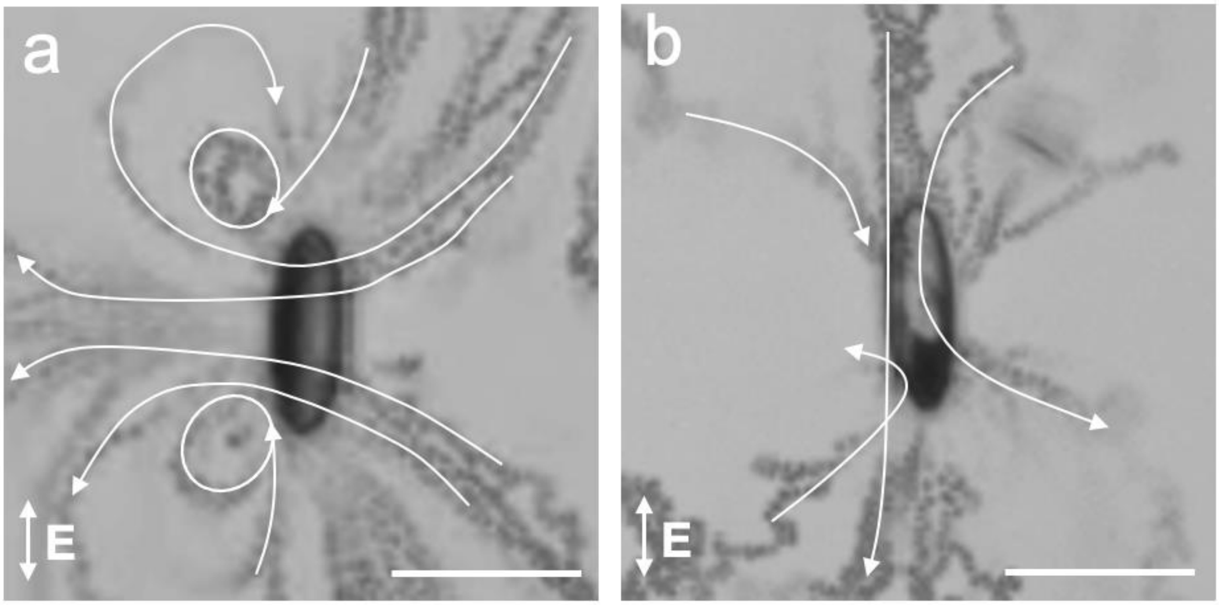

Figure S4. Superimposed images showing fluid flow profile around metallodielectric ellipsoids with the Janus patch (a) and the patch of lower symmetry (b). The arrows on the images indicate the trajectory of each tracer. Scale bars are 10 μm.

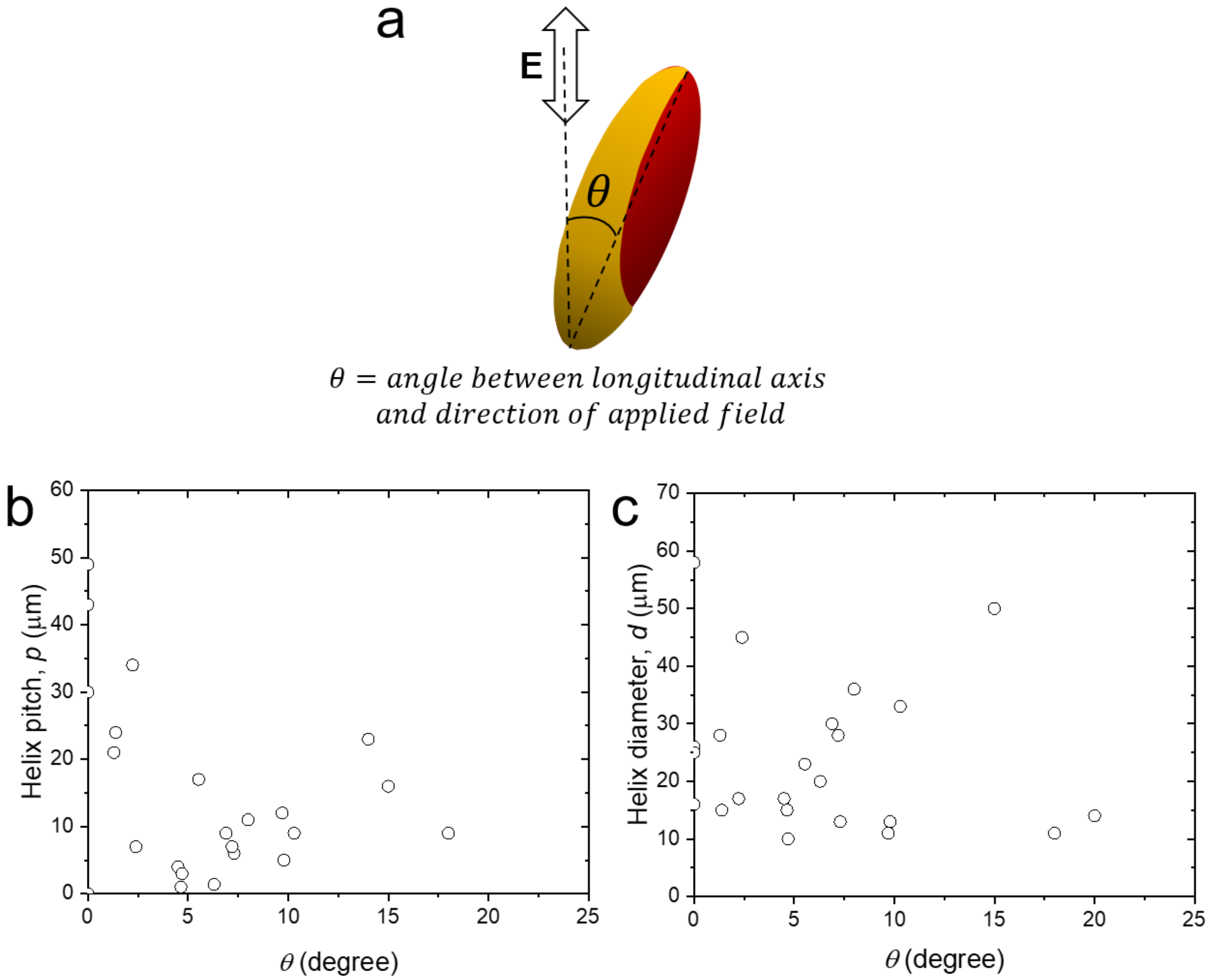

Figure S5. Alignment of self-propelling patchy ellipsoids in an electric field. (a) Schematic showing the definition of ellipsoid alignment angle (θ). The arrow indicates the direction of applied field. (b-c) Helix pitch (b) and diameter (c) at corresponding θ. The alignments of most ellipsoids are distributed at $\theta = 5 \pm 5°$ and show no clear relation with trajectories.

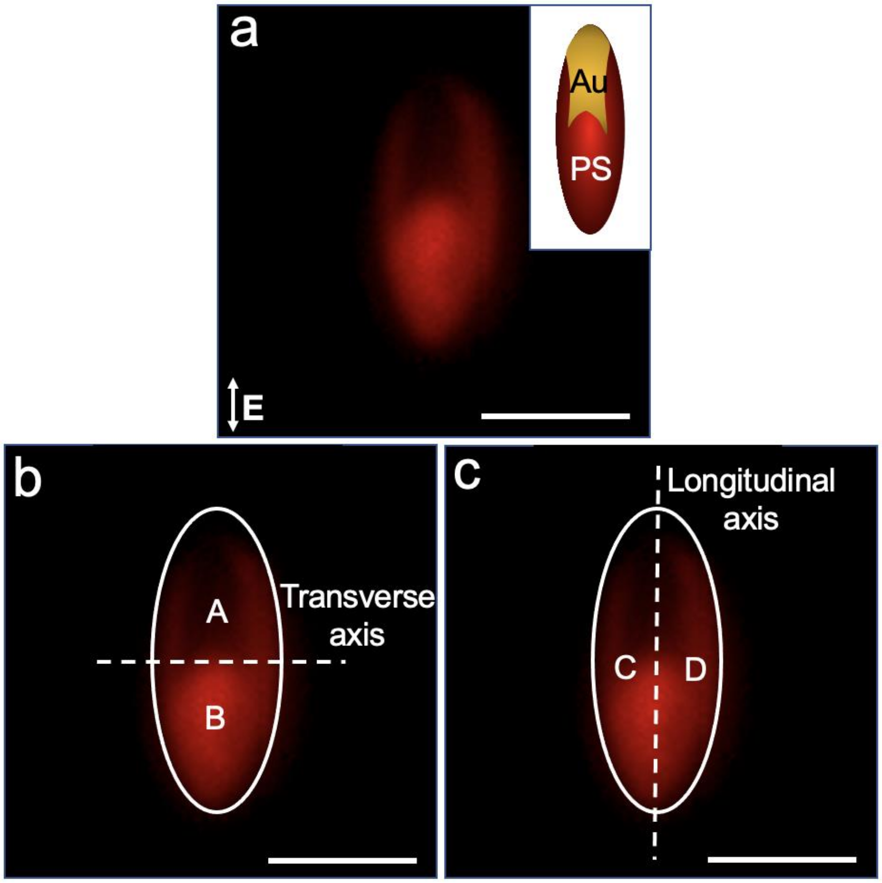

Figure S6. Fluorescence microscope images describing the patch ratio along the transverse axis (b) and longitudinal axis (c) of an ellipsoid. The metal patch appears as black on the red PS surface. Each active ellipsoid is divided into two regions by the ellipsoid's transverse or longitudinal axis. The larger patch area is assigned as "A" or "C", and the smaller patch area is assigned as "B" or "D". The symmetry parameters, ϕ and χ, are calculated as the ratio of metal patch between two regions of an ellipsoid. The inset in (a) is the schematic of the patchy ellipsoid. Scale bars are 5 µm.
